# Patterns of musculoskeletal growth and dimensional changes associated with selection and developmental plasticity in domestic and wild strain turkeys

**DOI:** 10.1002/ece3.3881

**Published:** 2018-02-19

**Authors:** Kristin K. Stover, Daniel M. Weinreich, Thomas J. Roberts, Elizabeth L. Brainerd

**Affiliations:** ^1^ Department of Ecology and Evolutionary Biology Brown University Providence RI 02912 USA

**Keywords:** allometry, morphological evolution, phenotypic plasticity, selection—artificial

## Abstract

Domestication is a type of experimental evolution in which humans have artificially selected for specific desired traits. Selected strain animals can be utilized to identify correlated responses by comparing them to the wild strain. In particular, domestic turkeys have been selected for increased body mass and high‐growth rate, most significantly over the past 60 years. Yet it remains unclear how artificial selection has affected the morphology and evolution of the musculoskeletal system as a whole. Here, we compare growth rate over 21 weeks, hind limb bone scaling across ontogeny via in vivo CT scanning, and muscle proportions in wild and domestic turkeys to identify differences in structural scaling and the potential contributions of selection and developmental plasticity to whole‐organism morphology. The domestic turkeys grew at a higher rate (0.14 kg/day vs. 0.05 kg/day) and reached over 3 times the body mass of wild birds. Comparing the proportional muscle masses in adult turkeys, only the trunk had a greater mass ratio in the domestic turkey, driven solely by *M. pectoralis* (2.8 times larger). The proportional increase in only breast meat and no other muscles highlights the surgical precision attainable with artificial selection. The domestic turkey femur and tibiotarsus displayed increases in polar moment of area, apparently maintaining torsional strength as body mass increased. The lack of dimensional change in the more vertically held tarsometatarsus is consistent with the pattern expected due to developmental plasticity. These results from the domestic turkey emphasize that there are morphological limits to preserving the balance between growth and function, and varying rates of trait evolution can further complicate this equilibrium.

## 1 INTRODUCTION

**Figure 1:**
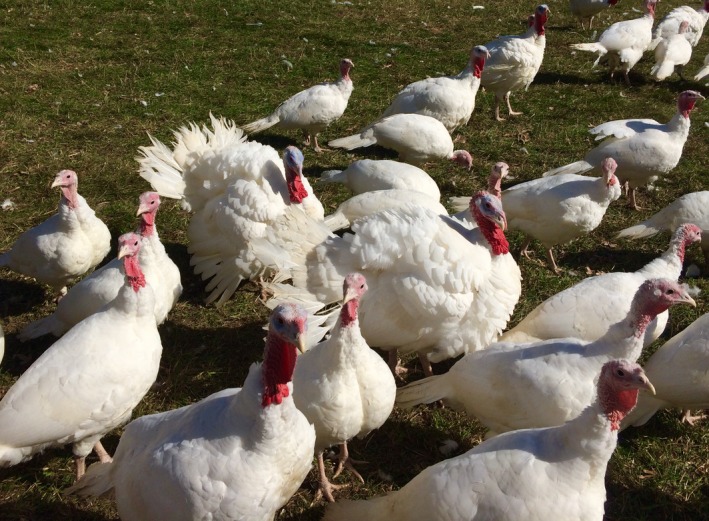
One of the two turkey strains used in this study, the broad‐breasted white domestic turkey

Artificial selection on domestic species can be very strong, resulting in animals that are quite distinct from their wild ancestors (Larson & Fuller, 2014; Lega, Raia, Rook, & Fulgione, 2015; Trut, Oskina, & Kharlamova, 2009). Domestic turkeys are one example of an animal bred to reach a much higher body mass than their wild counterparts (Figure 1). Over the past 60 years, the poultry industry has cut the time to market in half for domestic turkeys while increasing their body mass by twofold (Barbut et al., 2008). Selection by commercial turkey producers is accomplished through a breeding pyramid, often involving four different pedigree lines, each with distinct trait objectives (Neeteson, McAdam, Swalander, & Koerhuis, 2016). Traits that are under selection in various lines include growth rate, age to market weight, improved breast meat yield, feed efficiency, egg production, fertility, and hatchability (Anthony, 1998). However, various health concerns have been associated with this intense selection for traits like increased body mass, including skeletal deformities such as tibial dyschondroplasia and muscle pathologies such as white striping and deep pectoral myopathy (Julian, 1998; Kuttappan, Hargis, & Owens, 2016; Wilson, Nieberg, Buhr, Kelly, & Shultz, 1990). These problems arise from a variety of genetic, metabolic, and structural issues associated with selection for increased body mass in the domestic turkey.

As animals increase or decrease in size through evolution, not all structures scale the same. This can lead to functional limitations as some anatomical structures slowly catch up to those that change the fastest. The teeth of humans and dwarfed descendants of hippos are prime examples. Tooth size has not decreased as quickly as jaws have become smaller, therefore displaying negative allometry of the jaw relative to the teeth (Gould, 1975; Shea & Gomez, 1988). During domestication, direct selection for a trait, such as increased body mass, may accelerate changes for that particular trait, while others lag behind. In addition, differences in trait heritability can also widen the scaling disparity. In light of this, it is quite possible that a mismatch in structure size could be present in the relatively recently domesticated turkey. While artificial selection has increased overall body mass in domestic turkeys, individual muscle proportions may be different from those of wild turkeys. Scaling differences in the domestic turkey's morphology could cause or exacerbate many of the health problems that have developed. In addition, the scaling of the skeletal frame supporting the increased body mass must be undergoing modifications in morphology to maintain functionality. Developmental plasticity, on the other hand, may play a major role in fine‐tuning structural scaling to maintain function. Wild and domestic turkeys may be a suitable model for investigating coordinated evolution of the musculoskeletal system and the degree to which plasticity plays a role in maintaining function.

Here, we present a common‐garden growth study on wild and domestic turkeys, including both comparisons of mature birds and longitudinal data on skeletal growth from in vivo computed tomography (CT) scans. Our first aim is to establish growth curves for both strains of turkey to better understand how domestic turkeys achieve their greater adult body mass: through a faster growth rate, a longer growth period, or some combination of the two. Based on the selection for growth rate, we hypothesize that the domestic turkeys will achieve higher body mass by growing more rapidly than the wild turkeys. Second, we aim to describe how the hind limb bones of the two strains differ in relative dimensions throughout ontogeny. If hind limb bone dimensional changes are solely due to heritable genetic changes that accompany the selection for increased body mass, we might expect to see modifications in the three limb bones’ dimensions that are similar in magnitude and allometric direction during ontogenetic growth. With phenotypic plasticity, we would expect to see differences in bone dimensions among hind limb bones that experience different loading regimes. More specifically, we would hypothesize that the bones held more horizontally would be subject to larger dimensional changes due to high bending and torsional loads. The third aim is to determine whether all muscles in the domestic turkey have experienced the same proportional increase in size compared to the wild turkey. We hypothesize that all muscles will have increased in mass by the same relative amounts under selection for increased overall body mass in the domestic turkey.

We seek to understand how the morphology of muscles and bones has changed in the domestic turkey with increased body mass. If the coevolution of bone and muscle dimensions can be understood, then traits that are changing more slowly than the rest of the musculoskeletal system could be identified and selected upon more directly. This study can also give us insight into musculoskeletal changes associated with increased body mass in other organisms, such as how the human body responds to obesity. We can utilize these rapidly evolved turkeys to appreciate larger trends in the evolution of the musculoskeletal system, by describing how labile the system can be under known selective regimes of the commercial poultry industry (Neeteson et al., 2016).

## 2 METHODS

### 2.1 Animals

Eastern wild strain (females *n* = 4, males *n* = 2) and broad‐breasted white strain (males *n* = 10) turkey poults, *Meleagris gallopavo*, were obtained 2 days posthatch from licensed breeders and housed in the Animal Care facilities at Brown University in the summer of 2013. Four more male wild turkeys were raised in the summer of 2014 to increase the sample size for males. All turkeys previously mentioned were used to establish growth curves and bone growth patterns via CT scans. The mass of each turkey was recorded at least once and up to six times per week to establish a growth curve, until it began to plateau. In addition, six female domestic adults were obtained in October 2014 from a local farm, raised on pasture. The adult females were obtained to supplement the adult turkey numbers for determining muscle proportions in the domestic birds. Turkeys raised in the Animal Care facilities were maintained on an ad libitum water and 28% protein commercial poultry diet for the first 8 weeks and then transitioned to regular poultry feed. Both strains were raised together in a common pen environment.

### 2.2 Computed tomography scanning

Wild and domestic turkeys were anesthetized every 2 weeks until 14 weeks old, when girth became an issue with the rotating C‐arm, to collect in vivo CT scans for the longitudinal data set on hind limb skeletal growth. Scans were performed with a veterinary, cone‐beam CT scanner (Fidex CT Scanner, Animage LLC) with an X‐ray power of 120 kv and 60 mA, a slice thickness of 0.34 mm, and a standard reconstruction. A total of 121 CT scans were taken and analyzed for the 2‐ to 14‐week longitudinal series (5–10 individuals per strain per age class). Each bird was scanned a maximum of seven times with an exposure time of 15–30 s for each part of the body. The Animage Fidex CT scanner is an in vivo veterinary CT scanner designed for safe scanning of companion animals. We observed no acute effects of the X‐ray exposure and no obvious cumulative effects. We compared CTs from mature birds that had not had prior scans to those that had multiple scans, and noticed no qualitative differences in overall bone shape or robustness. All longitudinal scans were performed when turkeys were anesthetized with isoflurane O_2_ mixture (approximately 400 ml O_2_ per minute and 0.75%–1.5% isoflurane) via a mask that enclosed the head.

### 2.3 Skeletal measurements

Morphological measurements from the CT scans of the hind limbs were taken to compare growth rates between the two strains. Length measurements of the limbs could be made directly in the Fidex workstation scanning software using the measure tool. The cross‐sectional area (CSA), minimum, and maximum second moment of area on the hind limb bones were measured at the mid‐diaphysis using the Slice Geometry tool in BoneJ (Doube et al., 2010). The polar moment of area (PMA) was calculated with the equation:

(1) $$\text{PMA}=I_{\min}+I_{\max}$$

where *I*_min_ and *I*_max_ are the second moments of area as measured in BoneJ. Both the second moment of area and the PMA are measures that account for both the amount of material and how it is distributed around a neutral bending axis, with the highest stresses on the surface. Distribution of bone further from the axis of bending increases the PMA and influences the bone's strength in bending and torsion. The second moment of area characterizes the bone's ability to resist bending, while the PMA is used to estimate torsional strength (Wainwright, Biggs, Currey, & Gosline, 1976). PMA is thought to be a more realistic measure of long bone strength because it accounts for the axial and rotational forces acting on the bone, which are more likely to cause failure than simple bending (Lieberman, Polk, & Demes, 2004).

A prediction of domestic turkey hind limb bone length was also calculated by assuming isometric scaling from wild turkey dimensions. The average mass of the domestic turkey was divided by the average wild turkey body mass at each time point. This value was raised to the 1/3 power to convert to length and then multiplied by the average wild turkey bone length at that age.

### 2.4 Muscle distribution measurements

Six adult turkeys per strain, three males and three females, were dissected to obtain muscle masses. All dissected turkeys were a subset of those used in the growth portion of the study except the three domestic females. Muscles were dissected out at their origin and insertion points and weighed, with the tendons and aponeuroses included. Muscles were grouped by anatomical location, rather than function, to determine how regional mass distribution might affect the CoM position. The regions included the forelimb (*mm. biceps brachii, scapulotriceps, humerotriceps,* and *coracotriceps*), the trunk (*mm. latissimus dorsi caudalis, supracoracoideus,* and *pectoralis*), the proximal hind limb (*mm. flexor cruris lateralis, iliofibularis, iliotibialis lateralis,* and *cranialis*,* femorotibialis*, and *iliofemoralis*), and the distal hind limb (*mm. peroneus, gastrocnemius lateralis and medialis,* and *tibialis cranialis*). Because of the interest in regional mass, we selected some of the most massive muscles from each body region, as well as muscles from each group that performed a range of functions (flexion, extension, adduction, abduction). Therefore, relatively small muscles, such as the digital flexors, were not included. The sums of the muscle masses in each body region were divided by the total body mass to obtain a muscle mass to body mass ratio. The muscle group masses were also divided by the tibiotarsus length^3^ to address skeletal dimensional changes between strains.

### 2.5 Statistics

The SMATR package in R was used to perform standard major axis (SMA) regressions using the log‐transformed data to compare the allometric relationships between the bone measurements and body mass, as well as the expected geometric slopes (Falster, Warton, & Wright, 2006; Warton, Wright, Falster, & Westoby, 2006). SMATR uses a likelihood ratio test comparing it with a chi‐squared distribution to test for common slopes and shifts in elevation using the Wald statistic. If no common slope is found between the groups, then a post hoc pairwise comparison was performed.

For the muscle mass ratios, a least squares fit analysis of variance was used to compare the wild and domestic groups of turkeys in JMP Pro 12.01. Holm–Bonferroni corrections were made for the number of muscles analyzed when comparing individual muscle masses and mass ratios, as well as for the bone measurement statistics. Both strain and sex were included in least squares fit analysis of variance as effects for the normalized muscle masses and body region ratios. The effects of sex on muscle masses normalized by body mass were tested and found not to be significant for any group, so sex was removed to increase power.

## 3 RESULTS

### 3.1 Turkey growth

**Figure 2:**
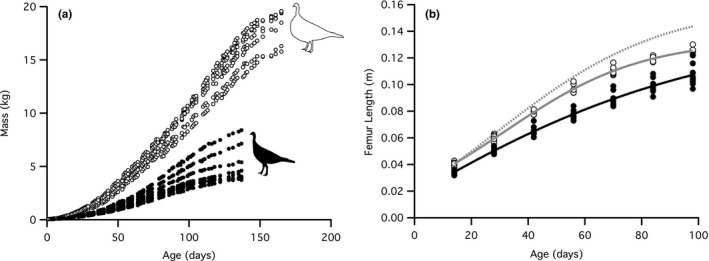
Body mass and femur length growth curves in wild and domestic turkeys. (a) Mass growth curves for domestic turkeys (open markers, *n* = 8) and wild turkeys (closed markers, *n* = 8) grown in the animal care facility from 2‐day‐old poults. At the peak of their growth, the domestic turkeys gained 0.14 kg/day on average while the wild turkeys gained 0.05 kg/day. The breaks in the data are days when the birds were not weighed, such as weekends. (b) Femur length growth curves from 121 in vivo CT scans taken every 2 weeks for domestic turkeys (open markers, gray line, *n* = 5–10 for each age group) and wild turkeys (closed markers, black line, *n* = 8–11) fit with the Gompertz equation. The femur lengths between strains are significantly different at all ages (*p* ≤ .0156). The dotted gray line indicates the expected isometric scaling of the length of the domestic turkey femur based on the increase in body mass over time, with actual lengths substantially lower

The domestic turkeys grew 2.8 times faster than the wild turkeys at the peak of their growth rate, measured at the midpoint of each curve (Figure 2a). The growth curves were sigmoid in shape, fit with the Gompertz equation, as previously described for high‐growth turkey lines (Anthony et al., 1991). The domestics reached a mean body mass of 18.4 kg (day 165) and the wild turkeys reached 5.3 kg (day 137). This makes the domestic turkey body mass over three times greater than that of the wild turkeys. The male turkeys of both strains were generally larger than the females; subsequently, sexual dimorphism explains a good portion of the variation among individual growth curves within each strain (Figure 2a).

### 3.2 Hind limb bone dimensions

**Figure 3:**
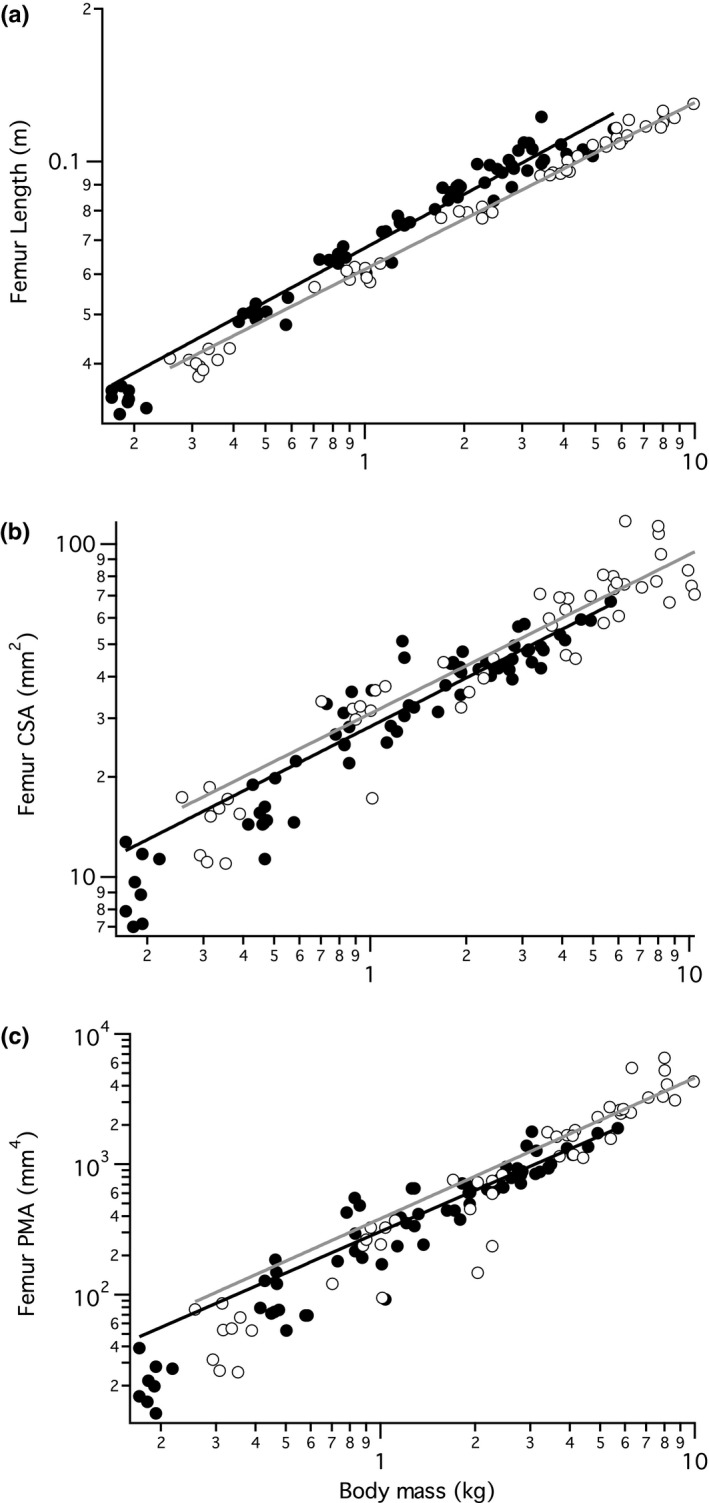
Scaling of the femur relative to body mass, from in vivo CT scans taken every 2 weeks for 14 weeks. (a) The length of the femur for wild (closed circles, black regression line) and domestic (open circles, gray regression line) turkeys across body mass. The scaling factor (slope) for the wild turkeys is M^0.38^, which is statistically significantly higher than the domestic scaling factor M^0.34^ (statistical results in Table 1). (b) Cross‐sectional area for the femur (mid‐shaft) across body mass, with non‐significantly different scaling factors of M^0.61^ and M^0.55^, respectively. (c) The polar moment of area for the femur, with non‐significantly different scaling factors of M^1.38^ and M^1.39^, respectively. The tibiotarsus and tarsometatarsus have similar scaling relationships (Table 1). All data are plotted on logarithmic axes

The scaling of the length, CSA, and PMA with body mass for the femur, tibiotarsus, and tarsometatarsus were compared using measurements from longitudinal in vivo CT scanning from weeks 2–14. Relative to wild turkeys, the growth in mass of the domestic turkeys outstripped the growth in hind limb bone length. The growth curve for femur length in the domestic turkey did not increase as quickly as the predicted curve based on the length of the wild turkey's femur with the increase in body mass (Figure 2b). Across ontogeny, the scaling exponents for the lengths of domestic turkeys’ hind limb bones were all significantly less than the scaling exponents for the wild turkeys (Figure 3a, Table 1). The result is that for any given body mass, the domestic turkeys had shorter hind limb bones. Or, to look at it another way, for any given bone length, the domestic turkeys carried greater mass (Figure 3a).

**Table 1:** Results from bone dimension SMA regressions

Bone	*n*	*R* ^2^	Slope	*P* strain	Shift^a^	Lower 95% CI	Upper 95% CI	Intercept	Lower 95% CI	Upper 95% CI	*H*_o_ Slope	*PH*_o_
Femur												
Domestic length	49	.991	0.340	.001^b^		0.331	0.350	−1.219	−1.224	−1.213	0.33	.036
Wild length	68	.97	0.382			0.366	0.399	−1.177	−1.183	−1.170	0.33	<.0001^b^
Domestic CSA	49	.898	0.555	.132	S	0.505	0.610	1.443	1.411	1.475	0.67	.001^b^
Wild CSA	69	.902	0.609			0.564	0.658	1.423	1.403	1.442	0.67	.04
Domestic PMA	51	.931	1.391	.849	S	1.289	1.500	2.333	2.269	2.397	1.33	.243
Wild PMA	69	.911	1.377			1.280	1.480	2.378	2.336	2.420	1.33	.346
Tibiotarsus												
Domestic length	49	.994	0.371	.001^b^		0.363	0.380	−1.040	−1.046	−1.035	0.33	<.0001 b
Wild length	68	.968	0.415			0.397	0.434	−0.984	−0.992	−0.976	0.33	<.0001^b^
Domestic CSA	51	.898	0.612	.768	S	0.558	0.670	1.376	1.342	1.411	0.67	.102
Wild CSA	69	.934	0.623			0.585	0.663	1.344	1.328	1.386	0.67	.069
Domestic PMA	51	.947	1.493	.406	S	1.398	1.595	2.128	2.068	2.188	1.33	.001^b^
Wild PMA	69	.94	1.438			1.354	1.526	2.166	2.130	2.202	1.33	.012
Tarsometatarsus												
Domestic length	49	.991	0.371	.001^b^		0.361	0.382	−1.173	−1.180	−1.167	0.33	<.0001^b^
Wild length	68	.972	0.412			0.395	0.429	−1.121	−1.128	−1.113	0.33	<.0001^b^
Domestic CSA	51	.845	0.535	.572	E	0.478	0.599	1.349	1.312	1.386	0.67	<.0001^b^
Wild CSA	69	.877	0.557			0.511	0.607	1.294	1.274	1.314	0.67	<.0001^b^
Domestic PMA	51	.925	1.217	.546	E	1.125	1.317	2.073	2.015	2.132	1.33	.028
Wild PMA	69	.91	1.177			1.094	1.266	2.010	1.974	2.046	1.33	.001^b^

Intercepts and slopes are based on a linear fit to the log‐transformed data, representing the “*a*” and “*b*” in the equation *y* = *aM* ^*b*^. *H*_o_ is the slope predicted by isometry.

a Shift indicated by WALD statistic, *E* stands for elevation shift with common slope, *S* stands for shift along common slope.

b Holm–Bonferroni Correction was used to determine rejection criteria.

**Figure 4:**
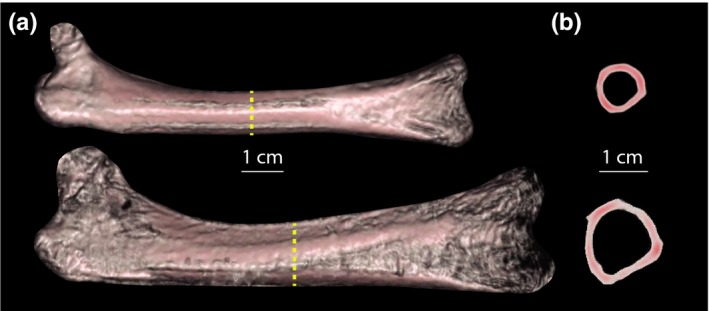
Femur length and cross‐sectional area for 14‐week‐old wild and domestic turkeys, from CT scans. (a) The wild turkey femur is from individual #48 (top) who weighed 3.49 kg and had a femur length of 10.6 cm. The domestic turkey femur (bottom) is from individual #8, who weighed 10.17 kg and had a femur length of 12.5 cm. The CT scans are left aligned to show relative lengths. The bone in the epiphyses of the domestic turkey femur appears to be less dense, as indicated by the holes near the distal ends of the bone. (b) Femur cross sections of the bones depicted in A, from a midpoint along the length of the bones (midshaft), approximated by the yellow dashed line. The domestic turkey femur is <20% longer than the wild turkey femur, but nearly twice the diameter

Polar moment of area was measured in the turkey long bones because it describes both resistance to torsion and bending loads. The scaling exponents for CSA and PMA with body mass were not significantly different between strains for the three hind limb bones (Figure 3b,c, Table 1). However, as the growth in length of the long bones in domestic turkeys was significantly slower relative to body mass (Figure 3a and Table 1), the domestic turkeys had a relatively higher CSA and PMA for a given length of bone, as a result of more bone being distributed further out from the central axis (Figure 4). The tarsometatarsus had a relatively lower PMA scaling exponent than the femur or tibiotarsus in both wild and domestic turkeys.

### 3.3 Muscle mass distribution

**Figure 5:**
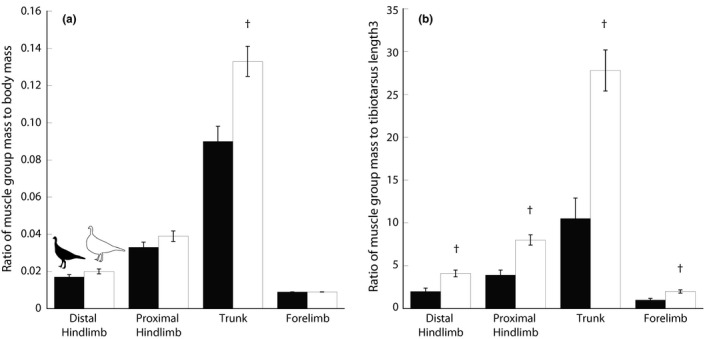
Masses of the regional muscle groups, normalized to body mass and tibiotarsus length cubed. (a) Muscle group mass normalized by total body mass for wild (filled bars, *n* = 6) and domestic (white bars, *n* = 6) turkeys. While all muscle groups increase in size with total body mass, the trunk group (driven by pectoralis muscle mass) makes up a larger proportion of the overall body mass in the domestic turkey. (b) Muscle group mass normalized by tibiotarsus length^3^ for wild and domestic turkeys. All body regions are significantly larger in the domestic turkey with respect to length of the long bones

The muscles were grouped by anatomical region, masses summed, and the total expressed as a ratio relative to total body mass (Figure 5a). The distal hind limb, proximal hind limb, and forelimb mass ratios were not significantly different between the two strains (statistical results in Supplementary Data, Table 1). Only the trunk had significantly greater relative mass in the domestic turkey, solely driven by *m. pectoralis* (Tables S2 and S3). We also normalized the muscle masses with length^3^ of the tibiotarsus to understand how the muscle mass was changing with respect to skeletal dimensions (Figure 5b). The tibiotarsus was chosen as representative of the long bones, but because we see a similar pattern of differences in bone lengths between strains in all of the long bones, we would expect the same results if we used the femur or tarsometatarsus to normalize. With respect to long bone length, all body regions of the domestic turkey are significantly more massive than in the wild turkey. Most individual muscles measured were about two times larger using this normalization method, with the exception of some muscles associated with the wing, including the *mm. biceps brachii, latissimus dorsi caudalis,* and the *scapulo‐, humero‐, and coracotriceps* that were not significantly larger than the wild turkey muscles. The trunk muscles are over 2.5 times larger in the domestic turkey than the wild turkey when normalizing to bone length^3^, again, driven by an extreme increase in the pectoralis muscle, 2.8 times the mass of the wild turkey pectoralis (Table S2).

## 4 DISCUSSION

### 4.1 Morphological changes associated with domestication

Our data indicate that domestic turkeys reach a larger body mass primarily by growing at a higher rate than wild turkeys, and to a lesser extent by growing over an extended period of time (Figure 2a). The impressive changes achieved by selection on domestic turkeys stem from the high heritability of body mass in these birds (Le Bihan‐Duval et al., 2003). On the other hand, the length of the skeletal elements has not kept pace with this increase in body mass, as shown by the difference between the femur length over time and the predicted isometric bone length curve (Figure 2b). Hence, the domestic turkeys are carrying around far more body mass on hind limb bones that are only slightly longer than the wild turkey bones. Hence, when normalized to body mass, the domestic hind limb bones are relatively shorter. These relatively shorter hind limb bones may serve to maintain stiffness, possibly helping to support increased body mass in the domestic turkey.

In addition to relative bone lengths, evidence from comparisons within and between the strains shows that bone radial dimensions change in a way that likely maintains strength as body mass increases (Table 1, Figures 3 and 4). The similar PMA scaling between strains suggests a structural response that maintains strength under the increasing load similar to the wild strain, although both strains scale slightly <1.67, the exponent expected for stress/strain similarity. The maintenance of PMA in domestic turkeys resulted from a relative increase bone diameter, as cortical bone was positioned more radially (Figure 4b). The radial placement of bony material is expected to be associated with greater stiffness and strength (Lieberman et al., 2004; Wainwright et al., 1976) of the hind limb bones that may help withstand the extra body mass of the domestic turkey. Our results are similar to those from a dimensional analysis study of canine radii which found that as body mass increased the bones became proportionally shorter, with tissue distributed more radially (Brianza et al., 2007). Changes in posture, such as becoming more upright, could also help compensate for increased muscle mass, but a previous study on domestic turkeys revealed that some strains are more crouched (Abourachid, 1993). One of the factors influencing maintained PMA scaling within turkeys is likely selection, as genotype and selection for certain hind limb parameters have been shown to affect bone thickness, length and weight (Damaziak et al., 2013; Emmerson, Anthony, Nestor, & Saif, 1991).

### 4.2 Responses to selection

Despite the apparent maintenance of leg bone strength with increasing mass, the domestic turkey shows poor locomotor performance with increased body mass and many leg health problems (Damaziak et al., 2013; Emmerson et al., 1991; Kapell, Hocking, Glover, Kremer, & Avendaño, 2017; Martrenchar, 1999). Body mass is known to be more heritable than leg health parameters in turkeys (Kapell et al., 2017; Swalander, Burnside, & Glover, 2012), so we find it somewhat surprising that the domestic turkey's bone strength, as indicated by PMA, keeps pace with the wild turkey and predictions from isometry (of course this could also be a plastic, developmental response, as addressed below). If the mineral density and material properties of the bone are consistent in the two strains, we expect the domestic turkey's bones to be just as robust for their mass as wild turkey bones. However, it is not likely that the wild and domestic turkey strains reach bone mineralization maturity at the same age, as seen in comparisons between domestic lines (Zhong et al., 2012). Indeed, during CT scanning we noticed that in many cases the long bone epiphyses of the domestic turkey seemed less dense (Figure 4a). Difference in bone material properties could contribute to fractures that are relatively common in the domestic turkey (Crespo, Stover, Taylor, Chin, & Shivaprasad, 2000).

Direct selection for increased pectoral muscle mass and greater overall body weight has been hypothesized to have caused these traits to increase at a faster rate than hind limb muscles (Nestor, 1984), and it has been extremely successful in this turkey strain. The only muscle that was significantly larger in domestic turkeys, when normalized to body mass, was the pectoralis superficialis (Figure 5, Tables S2 and S3). Wilson et al. (1990) also found that the pectoralis superficialis was relatively larger in the most rapidly growing lines of commercial turkeys they tested. Indeed, breast yield has only slightly lower heritability than overall body weight in turkeys (Le Bihan‐Duval et al., 2003). When we compare the ratios of muscle mass group to the length^3^ of the tibiotarsus, as another volume proxy that indicates size difference with respect to the skeleton, all of the body region masses were significantly larger in the domestic turkeys (Figure 5b). However, both normalization methods show that the increased muscle mass is not distributed uniformly, but rather is driven by the extreme pectoral hypertrophy. Another domestic fowl, the commercial broiler chicken, has relatively decreased pelvic limb musculature compared to Giant Junglefowl, their ancestor, and experience leg weakness disorders (Bradshaw, Kirkden, & Broom, 2002; Paxton, Anthony, Corr, & Hutchinson, 2010). The lack of relative increase in domestic turkey hind limb muscle mass with respect to the increase in body mass may contribute to leg weakness disorders. Weight distribution could also have a significant effect on balance and stability in large domestic turkeys (Abourachid, 1991, 1993) and could contribute to various issues related to leg weakness and gait (Nestor, 1984; Nestor & Anderson, 1998).

### 4.3 Developmental plasticity

The turkey was domesticated relatively recently, around 500–700 A.D., with the most intense selection for increased body mass and high‐growth rate occurring since the late 1800s (Dransfield & Sosnicki, 1999; Schorger, 1966; Smith et al., 2005; Yost, Kenney, Slider, Russell, & Killefer, 2002). This has lead to very rapid evolution of certain musculoskeletal structures; however, for some traits, it is difficult to determine which features can be attributed to heritable genetic changes (i.e., evolution) versus developmental plasticity. Both muscles and bones can be altered plastically within and animal's lifetime by responding to activity, resources and loading conditions. Local loading promotes bone remodeling (Wolff's law), which can induce significant changes to bone architecture such as the humeral hypertrophy seen in the playing arm of tennis players (Jones, Priest, Hayes, Tichenor, & Nagel, 1977). Skeletal muscles are also capable of responding to stimuli both in form and function by altering fiber phenotype, adjusting factors like fiber size or type (Flück, 2006; Pette, 2001). Our results indicate there is also some amount of developmental plasticity contributing to bone dimensions and muscle masses in the domestic turkey.

Data presented above provide compelling evidence that heritable, genetic effects are responsible for the relative differences in the size of the pectoralis muscle between wild and domestic turkeys. While we know much less about the relative contributions of genetic and developmental effects for other differences in morphology, our data are consistent with a substantial role for developmental plasticity influencing muscles associated with locomotion. Firstly, as a consequence of genetic variation influencing the pectoralis, we would expect developmental plasticity to cause the hind limb muscles to increase in relative size to support the extra body mass during terrestrial locomotion. Indeed, the hind limb muscles increase in relative size, when muscle mass is normalized to tibiotarsus length^3^ (Table S3). In contrast, as domestic turkeys have lost the ability to fly, we might not be surprised that forelimb muscle masses are unchanged compared to the wild turkey (Table S3). We acknowledge that it is also probable that there is genetic variability involved with the increase in hind limb muscle mass, but this cannot be discerned with our data.

The preservation of the domestic turkey's hind limb bone dimensions is also consistent with a plastic response. Bone remodeling has been shown to maintain similar strain levels throughout ontogeny in chickens (Biewener, Swartz, & Bertram, 1986), and turkey femur remodeling has been found to correspond with body weight (Zhong et al., 2012). If plastic bone remodeling in the heavy domestic turkeys contributed to their high PMA, we would expect to see the most pronounced changes in PMA in the bones that are subject to high bending and torsional loads. In other words, PMA should have a lower scaling exponent with body mass in bones that do not experience as much bending and torsion. Indeed, within both strains, the tarsometatarsus, which is held more vertically and probably encounters more shear and compressive forces (Loitz & Zernicke, 1992), had lower CSA and PMA scaling exponents than the femur or tibiotarsus, which have a more horizontal posture during most activities. These scaling differences among bones support the theory that certain bone radial dimensions are quite plastic, allowing alterations to maintain strength.

On the other hand, selection has also undoubtedly contributed to the maintenance of hind limb bone dimensions across ontogeny. Selection for hind limb function is made in each generation of commercially bred birds using gait scoring to select for turkeys with healthy walking dynamics, only breeding turkeys with hind limb morphology that enables effective locomotion (Garner, Falcone, Wakenell, Martin, & Mench, 2002; Kestin, Knowles, Tinch, & Gregory, 1992; Swalander et al., 2012). A combination of bone remodeling due to supporting the domestic turkeys’ increasing body mass during growth and selection for walking ability has probably lead to the conserved PMA dimensions we found in these birds. In mice selectively bred for running activity, changes in bone dimensions were attributed to both access to exercise and genetics (Wallace, Tommasini, Judex, Garland, & Demes, 2012). It is possible that in an effort to remodel the hind limb bone's dimensions to keep pace with the increase in mass, resources may be shifted away from maintaining bone density and mineralization. Selection for greater bone density combined with the observed bone PMA maintenance in domestic turkeys could help prevent fractures, similar to the decrease in keel bone damage seen in high bone strength chicken lines (Stratmann et al., 2016). X‐ray monitoring has been extremely successful in eradicating tibial dyschondroplasia (Swalander et al., 2012); the addition of hydroxyapatite mineral standards during screening would allow for a simultaneous measurement of 2‐D bone mineral density.

### 4.4 Plasticity and evolution

Interestingly, there are many examples of trait plasticity correlating with genetic lability in diverse systems (Crozier & Hutchings, 2014; Kelly, Czech, Wight, Blank, & Garland, 2006; Schlichting & Wund, 2014). Plastic traits may provide genetic pathways upon which natural selection can act (Draghi & Whitlock, 2012). Our results show that focused trait selection for white breast meat has resulted in a striking genetically determined increase in pectoralis muscle mass. In the wild, turkeys use burst flight mostly to roost or to escape predators, behaviors unknown to domestic turkeys, and yet this particular flight muscle far outpaces all other muscles in selective response. The pectoralis also undergoes large plastic changes in size in some birds, used as a reservoir for energy in migration and during seasonal starvation (Lindstrom, Kvist, Piersma, Dekinga, & Dietz, 2000; Piersma, Gudmundsson, & Lilliendahl, 1999). The flight muscles also contribute to shivering thermogenesis, an important heat production mechanism for many bird species, and a plastic increase in pectoralis mass is associated with increased thermogenic capacity (Petit & Vézina, 2014; Swanson, 2010). We speculate that the increase in pectoralis size may be an example of trait plasticity and genetic lability correlation. It is plausible that the same metabolic pathways used to plastically alter pectoralis size may also provide localities for genetic mutations upon which artificial selection can take advantage. Certainly, selection for increased pectoral muscle mass has also been very successful in the domestic chicken and duck (Farhat & Chavez, 2000; Zuidhof, Schneider, Carney, Korver, & Robinson, 2014), as well as a well‐documented plastic response for the same traits.

The association between pectoralis plasticity and heritable change, as discussed in the previous paragraph, suggests that highly plastic traits may be very effective places to also make genetic strides. Bones are able to plastically respond to loading by remodeling, which likely accounts for some portion of the maintained PMA in the domestic turkey. In light of this developmental plasticity, we suggest that there may be more room for implementing genetic modifications to increase hind limb bone strength by further exploring the mechanisms associated with bone remodeling.

## 5 CONCLUDING REMARKS

Our first aim was to establish growth curves for both strains of turkeys. We found that domestic turkeys achieve a greater adult body mass, three times that of the wild turkeys, both by growing at a faster rate and for a slightly longer period of time. Our second aim was to describe the hind limb bone dimensions across ontogeny. We found that domestic turkeys did not increase their bone length as quickly as we might expect with the increase in body mass, but CSA and PMA had similar scaling exponents between strains. Finally, we wanted to determine whether all domestic turkey muscles experience the same proportional increase in size compared to wild turkeys. We determined that the pectoralis muscle makes up a significantly greater proportion of body mass in the domestic turkey. These findings can inform our understanding of the processes that have contributed to the morphological changes associated with domestication in the turkey.

This investigation of wild and domestic turkey morphology has revealed how focused selection on a few traits can reshape an animal's musculoskeletal system through evolution and plasticity. The morphology of the domestic turkey is not what we would expect from simply scaling up a wild turkey isometrically. Instead, more weight has been added to an only slightly longer skeletal frame, and the pectoralis muscle has become relatively larger. However, the increase in PMA of the domestic turkeys’ hind limb bones, likely from a combination of plasticity and selection, may allow the bones to maintain strength as they support ever‐increasing amounts of body mass. Additional research on how these morphological alterations have affected the domestic turkey's hind limb posture and function is necessary. A recent study suggests that quantitative gait parameters, like step width, have a higher heritability than observational gait scores in poultry (Duggan, Rae, Clements, & Hocking, 2017). This study suggests that there may be more room for hind limb selection as well, by focusing on the highly plastic traits of the bones and muscles, as these types of traits have been correlated with evolutionary responsiveness. These results further emphasize that there are morphological limits to preserving the balance between growth and function, and varying rates of trait evolution can further complicate this equilibrium.

## DATA ACCESSIBILITY

The morphology measurement data sets and CT scans supporting this article can be accessed via the X‐ray Motion Analysis Research Portal at xmaportal.org, study ID BROWN21.

## CONFLICT OF INTEREST

The authors have no conflicting interests.

## AUTHOR CONTRIBUTIONS

K.K.S conceived the project, collected and analyzed data, and drafted the manuscript. T.J.R and E.L.B made contributions to design, data interpretation, and critical revision of the manuscript draft. D.M.W. contributed to data interpretation and manuscript revision. All authors gave final approval for publication.
